# The feasibility of an augment reality system to study the psychophysiological correlates of fear‐related responses

**DOI:** 10.1002/brb3.1084

**Published:** 2018-08-22

**Authors:** Susana Brás, Sandra C. Soares, Telmo Cruz, Tiago Magalhães, Bernardo Marques, Cláudia Dantas, Nuno Fernandes, José Maria Fernandes

**Affiliations:** ^1^ Department of Electronics, Telecommunication and Informatics (DETI) Institute of Electronics and Informatics Engineering (IEETA) University of Aveiro Aveiro Portugal; ^2^ CINTESIS.UA Department of Education and Psychology (DEP) University of Aveiro Aveiro Portugal; ^3^ Division of Psychology Department of Clinical Neuroscience Karolinska Institute Stockholm Sweden; ^4^ William James Research Center (WJCR) Instituto Superior de Psicologia Aplicada (ISPA) Lisbon Portugal

**Keywords:** augmented reality, fear, psychophysiology, specific phobia

## Abstract

**Background:**

Previous studies have successfully used augmented reality (AR) as an aid to exposure‐based treatments for anxiety disorders. However, to the best of our knowledge, none of these studies have measured the physiological correlates of the fear response, relying solely on self‐reports and behavioral avoidance tests.

**Methods:**

As the physiological defensive reactivity pattern impacts on the treatment effectiveness, we tested the feasibility of an AR system integrated in a mobile and wearable device for assessing the psychophysiological mechanisms (heart rate) involved in fear responses in real‐life contexts. Specific phobia was used as a model given its prototypical defensive hyperreactivity toward the feared stimulus (spiders to spider phobics, in the current study).

**Results:**

The results showed that the stimuli presented using AR were able to induce physiological alterations in the participants, which were specific depending on the stimulus type (fear or neutral) and on the participants’ level of spider fear (phobic and control group). These physiological correlates of the fear response were reflected both in the intensity of heart rate (in relation to the baseline) and in the time needed to react and recover after the stimulus exposure. Finally, we tested a theoretical model that showed that the physiological responses of phobic individuals when facing their phobic stimulus only explained its own data.

**Conclusions:**

We argue in favor of the system's feasibility at capturing and quantifying the physiological dimension of fear‐related responses, which may be of great value for diagnostic and treatment purposes in anxiety disorders, namely specific phobia.

## 1 INTRODUCTION

Mental health disorders are highly prevalent around the globe (~450 million) and result in significant impairments and malfunctioning in several domains of the individuals’ life (WHO, 2001). The chronicity associated with many mental health disorders and the corresponding need for long‐term treatments entails significant economic costs for society. In order to mitigate such burden, the development of more sustainable and efficient treatments should be endeavored.

Anxiety disorders are the most common mental health issue (circa 14.6%) and are highly comorbid with other psychiatric disorders, with excessive fear and anxiety representing the hallmark of this nosological classification (American Psychiatric Association, 2013). The emotion of fear marks environment events as significant to the individual (e.g., hazardous stimuli) and promotes the organization of adaptive responses to effectively cope with such events (e.g., Öhman & Mineka, 2001). These responses involve changes in subjective (e.g., dislikes), psychophysiological (increased heart rate, respiratory rate, skin conductance), and behavioral (avoidance) components (Lang, Greenwald, Bradley, & Hamm, 1993), which are dysfunctional in anxiety disorders. In order to capture a complete picture of the fear response, it is crucial that these dimensions are assessed collectively, both for diagnostic purposes and/or as indexes of the intervention's effectiveness. Although anxiety disorders are characterized by an increased activation of the autonomic nervous system (Martin, Ressler, Binder, & Nemeroff, 2009), individuals vary widely, within and over diagnosis, in their defensive reactivity patterns, namely psychophysiological, which then impacts on the treatment effectiveness (Lang, McTeague, & Bradley, 2016).

Given that psychophysiological measurements involve the use of expensive equipment (e.g., the Biopac System), most studies (particularly those assessing treatment efficacy (Botella, Bretón‐López, Quero, Baños, & García‐Palacios, 2010), use subjective measures (i.e., paper and pencil) to assess both the psychophysiological and behavioral dimensions of the fear response. In addition, most of the studies are run in the laboratory, in a highly impoverished setting, compared to the real contexts in which the individuals display their natural reactions to the emotional stimuli (e.g., potentially threatening animals, such as spiders). Consequently, fear responses may not reflect the emotional phenomena as it occurs in real life. Thus, it is essential to reproduce a naturalistic setting (Wilhelm & Grossman, 2010) in order to be able to observe the patient's natural responses to threatening stimuli. In order to capture these events, it would be necessary to employ an ambulatory/”on the field” monitoring approach, which would allow an in vivo assessment of the fear responses and enable higher ecological validity and, therefore, more reliable results.

Recent technological developments, such as biomedical sensors, smartphones, and wireless telecommunications, have been used to measure, communicate, and process information (Bras, Fernandes, & Cunha, 2013; Colunas, 2010; Ribeiro, Colunas, Marques, Fernandes, & Cunha, 2011). Mobile devices are becoming ubiquitous and may have an enormous potential in psychological studies. Such devices merge several important properties that have great potential: their cost, when compared to typical laboratory settings, for example (Lee et al., 2012), their ubiquity, allowing a better coverage of the target population. Moreover, they may provide valuable assessment and feedback, for instance, as a treatment outcome. As the interaction with the threatening events/stimuli is not always possible and/or is highly aversive to the individual, important additional developments in the treatment of anxiety disorders, using virtual and augmented reality (VR and AR), have also been allowing higher levels of immersion than any other currently available solutions (Baus & Bouchard, 2014; Clemente et al., 2010, 2014; Pausch, Proffitt, & Williams, 1997; Riva, Baños, Botella, Mantovani, & Gaggioli, 2016). AR is a sub‐area of VR in which computer‐generated stimuli are imposed on an existing real environment. AR systems introduce synthetic elements in order to enhance the participant's perception of the real world, allowing the merge of reality and virtuality (Milgram & Kishino, 1994). With AR, the virtual stimuli are presented while maintaining the participant's sense of presence, therefore enhancing reality, instead of replacing it (Berryman, 2012), which is the case in VR. While there are a few studies using VR in phobic scenarios (Baños et al., 2011; Krijn, Emmelkamp, Olafsson, & Biemond, 2004), the use of AR is almost inexistent and has been proposed using controlled and dedicated‐hardware setups (Bretón‐López et al., 2010). However, as in most studies, subjective measures are used to assess psychophysiological and behavioral (i.e., objective) measures, which undermines the study of defensive reactivity in a physiological dimension, particularly because the subjective and psychophysiological dimensions are not highly correlated (Lang et al., 2016). In this context, there is a clear need for a solution that is able to ecologically and easily collect objective and quantifiable data. Among the psychophysiological measures used to access the physiological dimension of fear, heart rate is one of the most commonly used in ecological settings, given that is easily accessed by new technologies (wearable sensors) and not highly prone to noise (compared with EDA, for example).

Given that phobias, namely spider phobia, are rather context specific and are highly prevalent in the general population (American Psychiatric Association, 2013), their study is the ideal candidate to assess the feasibility of using a mobile and wearable device for investigating the psychophysiological mechanisms involving fear and anxiety. In a previous work, we presented AWARE (Cruz, Bras, Soares, & Fernandes, 2015), a low‐cost, mobile, and augmented reality‐based setup to collect the individual's fear responses to stimuli. AWARE makes use of generic mobile devices as a portable and convenient platform for stimuli presentation and multimodal monitoring. In this study, our aim was to validate the AWARE system as a tool for the quantification of the physiological dimension (Heart Rate; HR, given that fear induces an increased activation in HR) of fear responses in a real‐life setting, thus increasing ecological validity in order to enable its use as a decision support system for psychology clinical practice. We expected stronger physiological responses (HR variation in relation to the baseline) for fear (e.g., spiders) than innocuous stimulus (e.g., apple). We also tested theoretical models regarding the reaction and recovery response patterns of highly spider fearful participants, compared to a control group, in response to their phobic stimuli (compared to fear, but not feared, and neutral stimuli) to assess their adequacy to the data prediction. We expected that one model would be able to predict the psychophysiological correlates of the phobic responses, which would endorse the feasibility of the AWARE system to capture the physiological correlates of fear responses.

## 2 METHODS

### 2.1 Participants

Twenty participants (16 females), with an age ranging from 18 to 54 years (*M* = 22.13; *SD* = 8.46), were recruited at the University of Aveiro, in Portugal. Participants were medication free and did not suffer from any mental or neurological illness. Participants filled a Portuguese version of the Fear of Spiders Questionnaire (SPQ) (Klorman, Weerts, Hastings, Melamed, & Lang, 1974), in order to evaluate their subjective fear of spiders. Participants were grouped according to their spider fear level (high and low fear), assessed by the SPQ questionnaire. In line with previous studies (e.g., Soares, Esteves, Lundqvist, & Öhman, 2009), those participants scoring above the 75th percentile were included in the high fear group as potentially phobic (henceforth called the phobic group; *N* = 4; Mean Score=19.00, *SD* = 0.00) and those scoring below 25th percentile were included in the control group (now called nonphobic group; *N* = 8; Mean Score=3.88, *SD* = 1.13). However, no formal diagnosis based in the DSM‐V (American Psychiatric Association, 2013) was performed.

Verbal and written informed consent was obtained from participants. Moreover, the study was approved by our institutional review board and followed the guidelines of the Declaration of Helsinki and the ethical standards of the American Psychological Association (APA).

### 2.2 The AWARE (Aware aWe‐inducing Augmented REality) system

**Figure 1:**
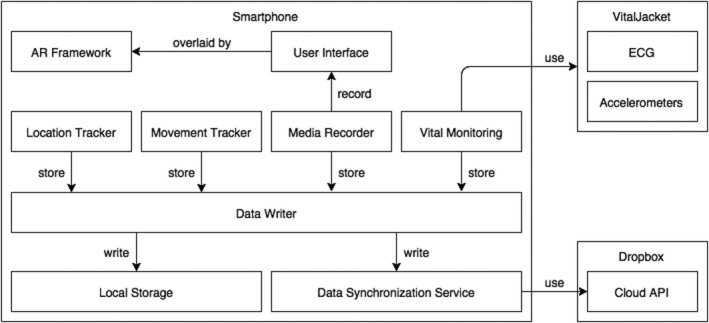
Architecture of the AWARE system. The Media Recorder component synchronously records the mobile's microphone and screen (camera input with AR overlay), in order to have the participant's own perspective for future studies, using the Data Writer component. Except for the media records (video and audio), the Data Writer stores, in the smartphone's storage. Whenever possible, the Data Writer's Synchronization Service uploads all data to the Dropbox Cloud based repository solution from—Dropbox, Inc waiting for an Internet connection in case of network absence

AWARE is an Android application able to synchronously monitor physiological and behavioral responses of individuals while they are presented with different stimuli. This application counts with both location and orientation sensors (GPS, accelerometer, and gyroscope), provided by the smartphone, to monitor behavioral reactions, as well as an autonomous vital monitoring wearable sensor (e.g., heart rate ‐ HR, and ECG wave) and enabling the collection of physiological responses. Additionally, the application overlays a camera input with an AR 3D model—the stimulus presented to the participant. AWARE was designed to be composed by several components (Figure 1) that interact with each other.

**Figure 2:**
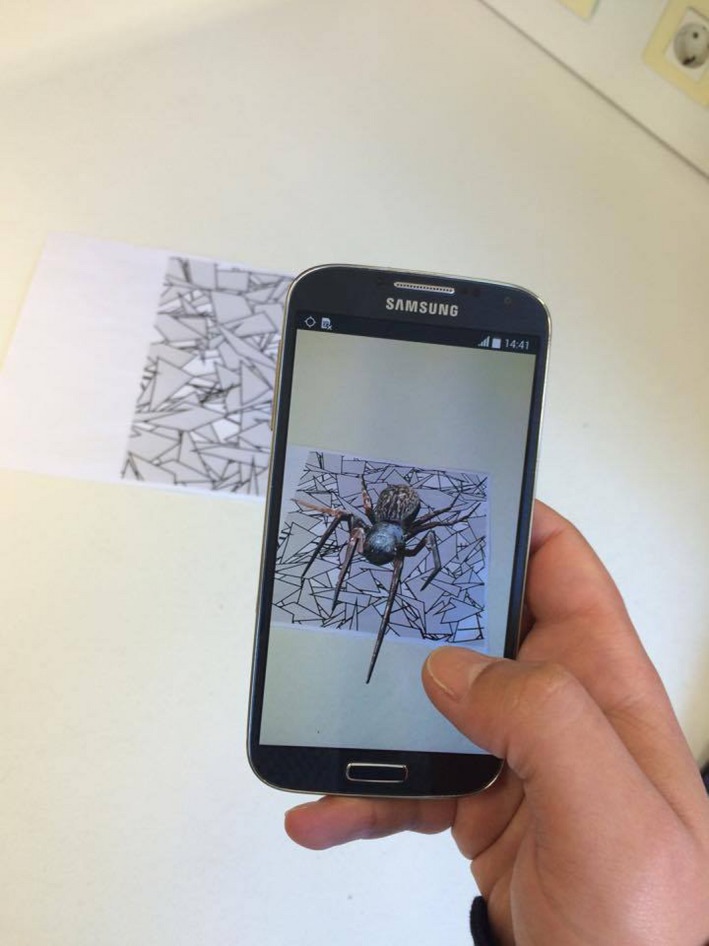
Illustration of the one participant using the smartphone as a magnifying glass and pointing it to an AR target (a spider, in this case)

During the tests, participants were asked to use the smartphone, as a magnifying glass, pointing it to a unique marker (Figure 2). This would enable overlay the synthetic stimuli 3D models as a camera input, displayed in the smartphone's participant interface. The unique markers were visually similar to the human sight, with the application being able to distinguish them using QCAR/Vuforia AR SDK (Augmented Reality framework optimized for mobile devices), from Qualcomm, Inc.^1^

For physiological monitoring, AWARE makes use of an external wearable device—VitalJacket by Biodevices, SA.^2^ This wearable system provides ECG raw data (1‐lead acquisition at 500 Hz), heart rate (HR), and RR (time interval between two consecutive R peaks) (Darell, 2011).

### 2.3 Experimental setup and protocol

Participants faced AR stimulus provided by the mobile enhanced view, integrated in a nonimmersive environment, hence taking advantage of AR. Each participant was exposed to 8 markers that triggered an AR stimulus provided by the mobile enhanced view. The stimuli set included three fearful (a gray spider and a white spider—potentially phobic for those participants scoring high in the SPQ and henceforth called phobic stimulus; and a cockroach, fearful but not reported as potentially phobic for neither of the participants, henceforth called fear stimulus) and five neutral markers (e.g., apple), that is, not expected to induce any strong responses by the participants. The lower number of fear stimuli was used to preclude anticipation effects. The order of the stimulus presentation was counterbalanced, and the markers’ sequence was randomly presented to each participant. The distance between markers was set at 5 to 10 meters to enable the measurement of the response to each stimulus and the subsequent recovery pattern. Each participant followed a marker trail twice. Physiological responses were collected throughout the experiment. A 1‐minute baseline was collected to characterize the physiological response pattern of each participant. Participants were then asked to follow the trail and touch each marker on the floor in order to ensure that the stimulus presentation was performed in a close‐range perspective.

## 3 RESULTS

### 3.1 Data analysis

In order to assess if the fear stimuli induced stronger physiological responses than the neutral ones, and whether the system captured sensitivity to habituation, we compared the HR data segments associated with different stimulus in the two marker trials. We used Wilcoxon rank‐sum test to compare the HR segments’ median and considered a *p* < 0.05, for the significance level, and Bonferroni correction for multi‐comparison analysis.

We built a theoretical model on the expected HR response and recovery for each participant after being exposed to a given stimulus. As high levels of fear are associated with stronger HR responses, particularly when participants are faced with their phobic stimuli, compared to feared but not phobic and neutral stimuli (see (Öhman & Mineka, 2001)), this model is ideal for investigating the psychophysiological correlates of the fear response as it aids the assessment of the deviation from other responses (not related with the model). Based on the expected responses in HR from the two groups (phobic and control) toward the different stimuli (phobic, fear but not phobic, and neutral), as well as the expected recovery response patterns, we tested the relation between models to infer the model's adequacy for the data prediction.

### 3.2 HR variation between stimuli

Heart rate variation (in relation to baseline) as a function of the stimulus category (fear or neutral) was calculated. Specifically, we used the maximum HR value of each stimulus and compared it with the baseline. Considering all the participants, independently of their level of spider fear, and as expected, we observed a significant difference in the medians between stimuli category (*p* < 0.001). More specifically, the median value for heart rate variation to baseline for the neutral stimuli was 4, and 6 for the fear stimuli, which indicates a higher reaction to the later stimuli, that is, to fear inducing stimuli (white spider, gray spider, and cockroach).

To study the sensibility of the system to capture habituation effects, we also assessed whether there were significant differences between runs, with the results showing significant differences for neutral stimuli between runs (*p* < 0.05), while no such effects were shown for fear stimuli (*p* > 0.05). Thus, no habituation effects were demonstrated for fear stimuli, which is a further demonstration of the feasibility of our system to capture specific HR responses to this type of stimuli.

#### 3.2.1 Reaction/Recovery

**Figure 3:**
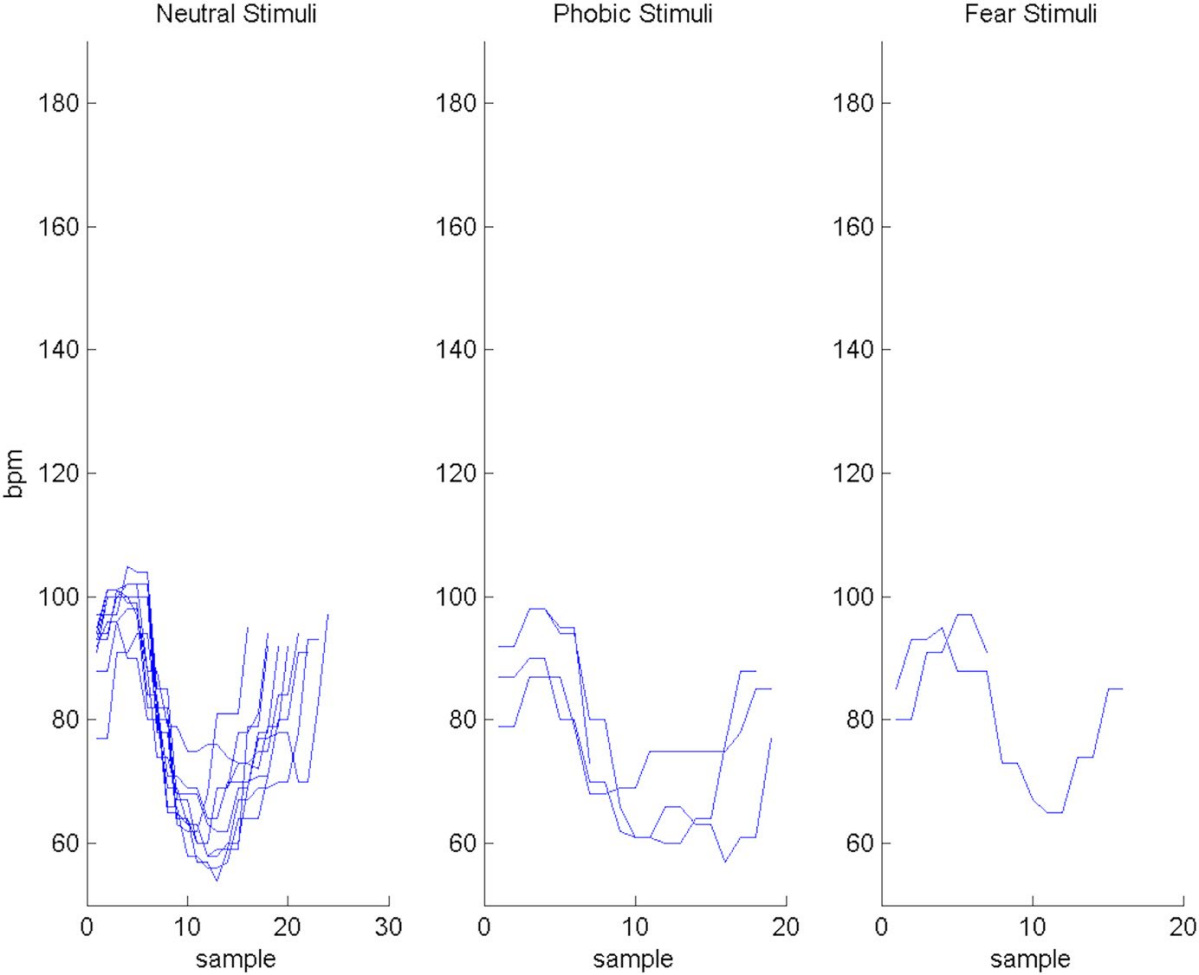
Heart rate (HR) response of participant 18 in both runs stimuli visualization. First, second, and third chart correspond to the response to neutral, phobic, and fear stimuli, respectively. Despite difference in intensity and duration when the participant faced any of stimulus (sample 0), it is possible to observe an increase in HR (response) and a decrease in HR afterward (recovery before sample 10)

**Figure 4:**
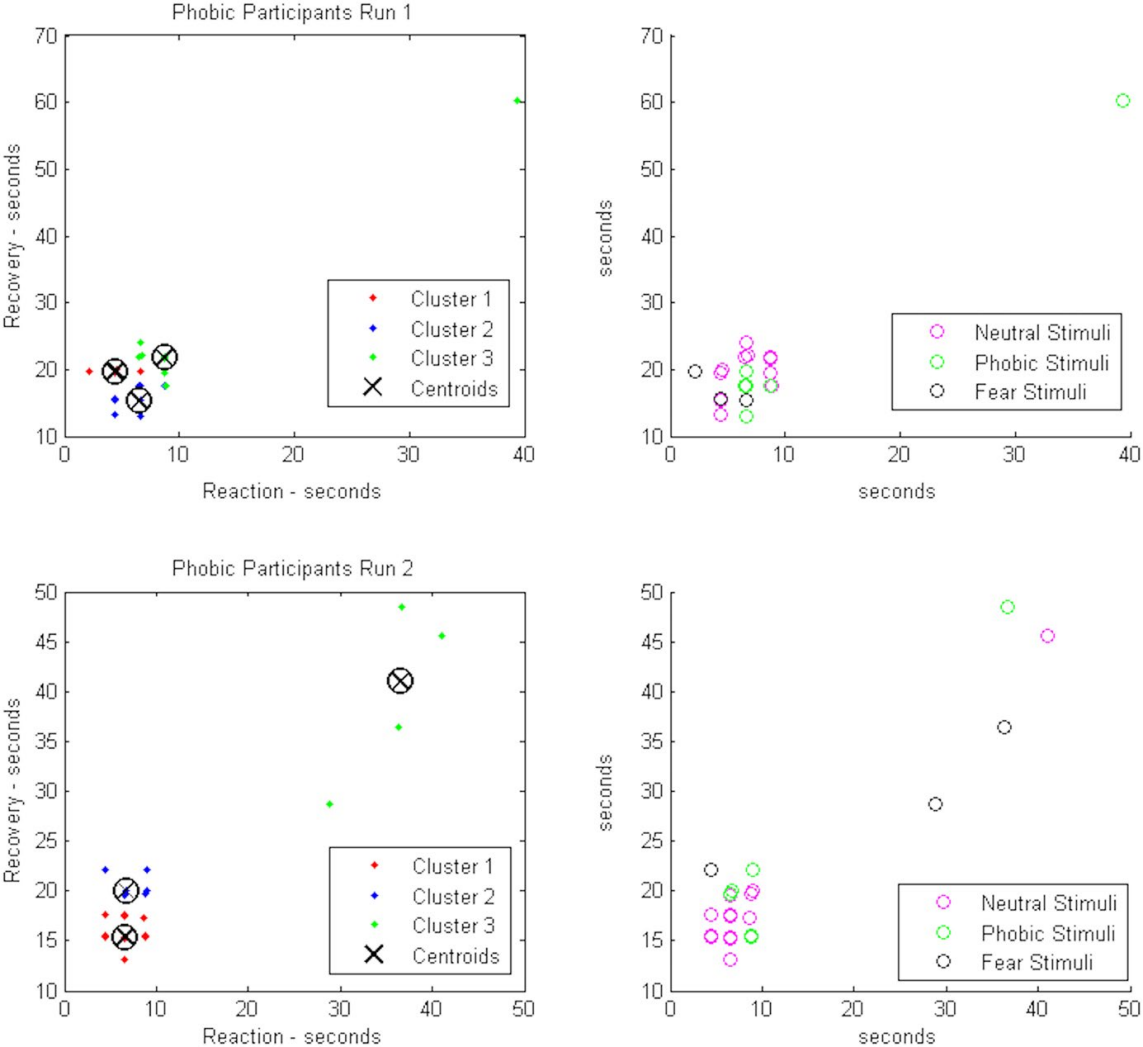
Phobic, fear, and neutral stimuli response vs recovery time scatters overlaid with automatic separation by k‐medians clustering algorithm for phobic participants

As depicted in Figure 3, when participants were exposed to a stimulus (sample 0), an increase in HR was expected (response), followed by a decrease in HR (a recovery, before sample 10). The intensity of these features, as well as their duration, provides important elements of the response pattern to stimuli of different categories (phobic, fear, and neutral). We considered the maximum point of HR as the point of reaction after the stimulus presentation, and the minimum value of HR the recovery point after the stimulus visualization to measure the time needed for each participant to react and recover after the stimulus presentation. These times were used to characterize each response‐recovery pattern and used later in a clustering system to assert if it was possible to differentiate responses according to different stimuli categories. In Figure 4, is represented the responses to the stimuli, more precisely, the relations between reaction and recovery time for each stimulus, for the phobic participants. We observed that a k‐medians algorithm (k‐medians uses the median values to estimate the centroid of the cluster), with three clusters, was able to automatically divide the data space in three groups. The identified groups may be associated to the stimuli category (fear, phobic, or neutral). Given the match between the points associated to each cluster and the points representing the stimuli, we observed that there were few points classified in the wrong group.

**Figure 5:**
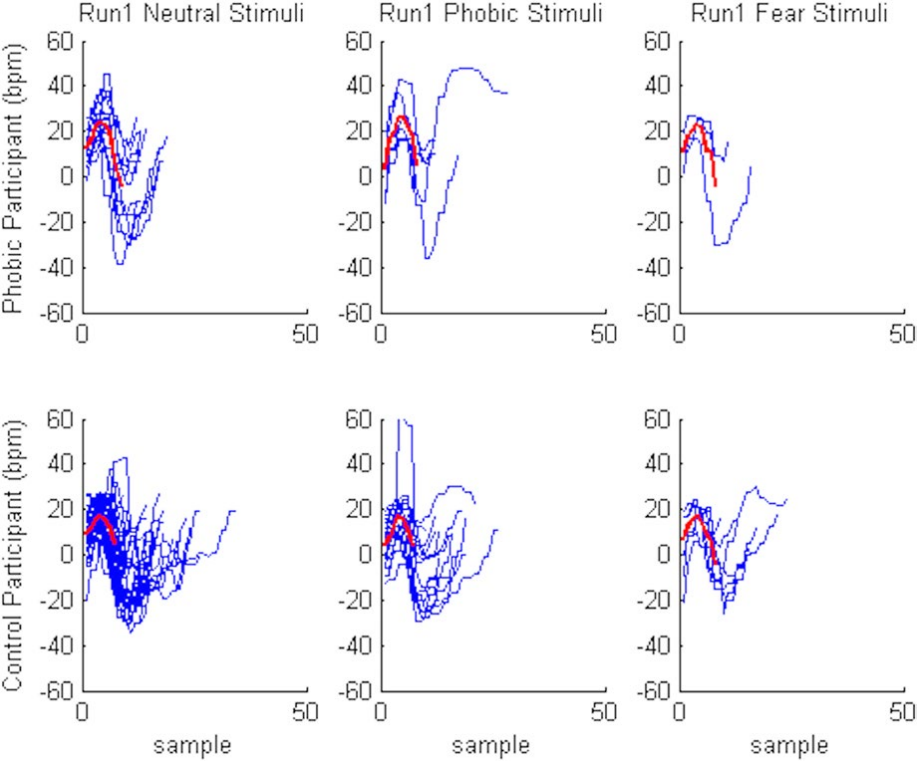
Theoretical model, that is, the expected response‐recovery (red) describing the participants’ response to the stimuli observation, superimposed with the individual responses to stimuli, evaluated by the HR variation to baseline (blue)

More importantly, we also aimed at investigating whether the participant's reaction was different when facing different stimuli categories and if this effect varied as a function of the participant's level of spider fear. As the first run corresponds to the first reaction to the stimuli (and therefore a more natural and realistic reaction), we designed a theoretical model that describes the average response of participants in each group (phobic and control group) and for each stimulus type (neutral, phobic, fear) in run 1, that is, the expected HR response‐recovery profile. Figure 5 presents the theoretical model in each case.

The model's adequacy to the data was inferred by the use of R^2^. When the model is unbiased, the R^2^ is between 0 and 1, where 1 corresponds to a perfect model fit. If R^2^ is negative (Mohseni, Stefan, & Erickson, 1998), the numerator is higher than the denominator and so the error deviation is superior to the signal variance. In this latter case, the model is biased and the measure is usually set to 0 (Norman & Streiner, 2008), meaning that the horizontal line at mean data value fits the data better than the model.

Evaluating the relation between models, that is, the explained variance (R^2^) of one model in relation with the other, allows the inference about the model's adequacy to the data prediction (Table 1). The results showed that in run 1, the model for neutral stimuli (e.g., apple) in phobic participants adequately described the data of other models in the same participants’ group. A similar result was observed in the model for the fear stimulus (i.e., cockroach) in phobic participants. Nevertheless, the model that describes the phobic stimuli in phobic participants (spiders, for the phobic group) was only able to describe its own data, as it did not explain the models in the other stimuli category. Regarding the control participants, the model found for one stimuli category was able to describe the other stimuli models. Notwithstanding this, the models for phobic participants could not be used to describe the data from the control participants, and the reverse was also true. This analysis indicates that the two groups of participants react differently, independently of the stimuli. Also, the phobic stimuli in the phobic participants revealed to explain only itself, indicating that it is characterized by a singular physiological pattern.

**Table 1:** Explained variance (*R* ^2^) between the theoretical stimuli response models. The zero value indicates biased models, that is, the horizontal line at mean data value fits the data better than the model

	Tested in:					
	Control Participant Neutral Stimuli	Control Participant Phobic Stimuli	Control Participant Fear Stimuli	Phobic Participant Neutral Stimuli	Phobic Participant Phobic Stimuli	Phobic Participant Fear Stimuli
Control Participant Neutral Stimuli	1.000	0.656	0.822	0.000	0.000	0.060
Control Participant Phobic Stimuli	0.447	1.000	0.593	0.000	0.000	0.000
Control Participant Fear Stimuli	0.787	0.698	1.000	0.000	0.000	0.000
Phobic Participant Neutral Stimuli	0.000	0.000	0.000	1.000	0.431	0.378
Phobic Participant Phobic Stimuli	0.000	0.000	0.000	0.008	1.000	0.000
Phobic Participant Fear Stimuli	0.000	0.000	0.000	0.480	0.262	1.000

**Figure 6:**
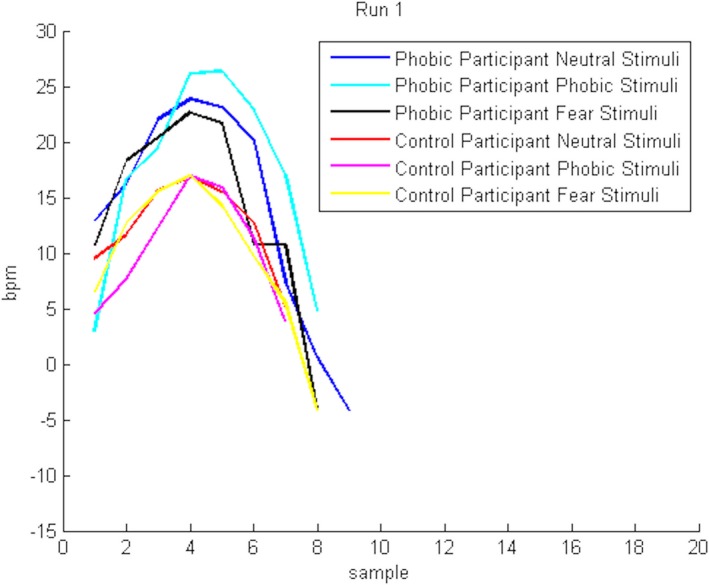
Theoretical models in run 1, describing all expected response profiles overlaid for the phobic and control participants’ response to neutral, phobic, and fear stimuli

The previously described results are confirmed in Figure 6, where we represent the six possible hypotheses (theoretical models, built from the collected data, by the average response of all participants to each type of stimulus) in run 1. In this run, the response pattern indicates that the phobic participants overreact to the stimuli, especially when facing the phobic stimuli (i.e., spiders).

## 4 DISCUSSION

The present study was designed to assess the physiological dimension of fear responses, in an ecological setting, through the analysis of heart rate (HR), given its easy assessment using current technology. To accomplish such goal, an Augmented Reality (AR) system incorporated in a mobile and wearable device was implemented for assessing the psychophysiological mechanisms involved in fear responses in real‐life contexts. We used specific phobia as the prototypical anxiety disorder regarding defensive hyperreactivity (McTeague, Lang, Laplante, & Bradley, 2011).

The assessment of the feasibility of the AR system incorporating HR responses was performed by building theoretical models that represent the response patterns by each group of participants, exposed to different stimuli (fear or innocuous). The results showed that the stimuli presented using AR, in an ecologically valid setting, could indeed induce physiological alterations in the participants. More importantly, these responses were different depending on the stimulus type (fear or neutral) and on the participants’ level of fear (phobic and control group) and were reflected both in the intensity of HR and in the time needed to react and recover after the stimulus exposure. The results indicated that the model that described the phobic individuals when confronted with their phobic stimulus only explained its own data and was not able to describe the fear responses in any other circumstance. Though, other models could describe each other; therefore, indicating that the responses were not different enough. This set of results is indicative of the system's feasibility at capturing and quantifying the physiological dimension of the fear‐related responses, which may be of great value for diagnostic and treatment purposes in anxiety disorders, namely specific phobic.

Notwithstanding the reduced number of participants in the phobic condition, the distinct and enhanced psychophysiological correlates of fear‐related responses captured by AWARE are consistent with the results from several studies run in highly controlled settings (i.e., laboratories). In particular, these studies have showed that when exposed to their phobic stimuli (e.g., spiders to a spider phobic), participants exhibit an enhanced activation of autonomic responses, as reflected by elevated skin conductance, startle responses and, more pertinent to the present study, hear rate acceleration (e.g., Carlsson et al., 2004; Globisch, Hamm, Esteves, & Öhman, 1999; Grillon, 2008; Öhman & Soares, 1994). As these pronounced psychophysiological responses to phobic stimuli are typically associated with intense subjective experiences of distress (for a review, see (McTeague et al., 2011), phobic individuals are often engaged in behavioral avoidance (for a review, see (Krypotos, Effting, Kindt, & Beckers, 2015). This coping strategy (i.e., behavioral avoidance) plays a critical role as a maintenance factor of the disorder (Craske, 2003), which is why exposure therapy is deemed as the most effective treatment strategy (Kaczkurkin & Foa, 2015), when compared to placebo and other treatment approaches, such as relaxation or cognitive therapy (Wolitzky‐Taylor, Horowitz, Powers, & Telch, 2008).

Exposure‐based treatments have indeed been pointed as an effective treatment in specific phobia by a substantial body of evidence (for a review, see (Davis, Ollendick, & Öst, 2012). Exposure interventions are rooted in classical conditioning, which assumes that the reduction of physiological responses occur through the repeated confrontation with the phobic stimulus (i.e., habituation), while behavioral and cognitive avoidance are prevented (see (Ougrin, 2011)). However, because in vivo exposure is reported as an extremely aversive experience, there is usually a high reluctance in engaging in therapy (e.g., Essau, Conradt, & Petermann, 2000), as well as a high number of dropouts for those who do initiate the therapeutic process (e.g., Garcia‐Palacios, Botella, Hoffman, & Fabregat, 2007).

Critical developments in the fields of virtual and augmented reality (VR and AR) have presented optimal solutions for counteracting the effects of in vivo exposure, namely by enhancing the patient's acceptance of exposure‐based treatments (see (Rizzo et al., 2010)). Both VR and AR systems allow high levels of immersion (Pausch et al., 1997), compared to other available solutions, such as Multimedia presentation which remove the sense of presence of a real world (Baus & Bouchard, 2014). AR superimposes synthetic computer‐generated stimuli on an existing real environment, enhancing the participant's perception of the real world (by merging reality and virtuality) and, therefore, increasing the sense of reality involved in the exposure. Importantly, the costs involved in the production of the environment are lower than in VR, which may increase the generalization of its use in clinical practice (for a review, see (Baus & Bouchard, 2014)). A few studies to date have employed AR techniques for stimuli delivery in anxiety disorders, namely in specific phobias such as spider phobia (Wrzesien, Burkhardt, Alcañiz, & Botella, 2011; Wrzesien et al., 2013) and cockroach phobia (C. Botella et al., 2011, 2010). In this later phobia, and after AR applied in a “one‐session therapy,” following the treatment protocol followed by (Öst, Salkovskis, & Hellström, 1991), the positive outcomes of the therapy persisted up to a 12‐month follow‐up period (Bretón‐López et al., 2010). However, none of these studies assessed the physiological dimension of the fear responses, relying solely on self‐reports to evaluate the treatment efficacy, such as fear and avoidance scales (e.g., Juan et al., 2005), subjective units of discomfort (e.g., Wrzesien et al., 2013), and the behavior avoidance test (e.g., C. M. Botella et al., 2005). Smartphones have been successfully used to collect and process psychophysiological data. Recent studies (Brás, Soares, Moreira, & Fernandes, 2015; Cruz et al., 2015) point these devices as being useful, portable, and inexpensive solutions to perform the required tasks and concomitant physiological assessment. Equally important, the results of the present study were successful in showing an AR system using such devices, even while recruiting participants without a formal diagnosis of specific phobia, although we recommend future studies to include such sample. Moreover, given the role of state anxiety in modulating fear responses, namely return of fear (Kuhn, Mertens, & Lonsdorf, 2016), future studies should also assess this variable in order to provide a deeper understanding of treatment efficacy and the mechanisms involved in relapse.

## 5 CONCLUSIONS

In the present study, we were able to show the feasibility of AWARE for physiological assessment while presenting AR 3D stimuli models in real‐life scenarios. The system involves a simplified logistic, which can be used in fear trigger prone stimuli and environments outside the laboratory. Importantly, the system may be adapted to different contexts and stimuli depending on the idiosyncrasies of each patient's therapeutic process and the specific anxiety disorder diagnosis. Future studies should also test the efficacy of the physiological dimension of the phobic responses collected by AWARE as treatment outcome in anxiety disorders.

## CONFLICTS OF INTEREST

The authors declare no conflicts of interest.

## Notes

1
https://www.qualcomm.com/

2
http://www.biodevices.pt/
